# Erie County Medical Society

**Published:** 1883-07

**Authors:** A. M. Barker

**Affiliations:** Secretary


### Erie County Medical Society.

The semi-annual of the Erie County Medical Society was held in the College building on the twelfth inst., the President, Dr. S. E. S. H. Nott, of Hamburg, in the Chair.

The minutes of the previous meeting were read and adopted. Dr. Dorland, chairman of the committee on membership, reported favorably regarding the following applicants and recommended that they be admitted to membership: Drs. F. H. Potter, Herman Hayd. J. W. Putnam, W. G. Gregory, E. H. Long, J. Wilmot Hunter and J. H. Pryor.

Applications for membership were received from Drs. R. A. Witthaus, William Meisburger, W. A. D. Montgomery, Burt. P. Hoyer, Benj. G. Long, Carlton R. Jewett, William H. Thornton, Claudius J. Niemand, Adelbert G. Gumar, Frederick W. Sweetland and Mary Berkes. They were referred to the committee.

Dr. H. R. Hopkins, as chairman of the special committee appointed to consider the passage of the bill establishing a State Board of Medical Examiners, presented the following preamble and resolutions:

Your committee upon which was laid the duty of urging the passing of the bill for the purpose of establishing a State Board of Examiners in Medicine, recently before our legislature, would report: That as soon as practicable after appointment, correspondence was instituted with the chairman of the committee on legislation of the New York State Medical Society, and with the members of your upper and lower branches of legislature. The result of this correspondence was to establish the probability of there being little chance for the bill passing at the present session, for the reason that the bill found a well-organized opposition, and upon its introduction in the senate was ordered before an adverse committee in which it was quietly strangled. At the time of the appointment of your committee, April 11th, it was too late to introduce new measures into the assembly such as the report of a committee. Your committee, in this forlorn condition, appealed to the Hon E. S Hawley, member of assembly from this district, and this gentleman took the bill before the committee on general laws and had a hearing in its behalf. The bill was favorably received by the committee, but owing to the lateness of the session, they were unable to bring it before the house, and the session closed without having a chance to seriously consider the bill.

Your committee have reliable information fixing the opposition to this bill upon prominent members of our own profession, and as would naturally be supposed upon men who are largely responsible for the evils which the proposed measure was designed to correct.

Your committee is aware that reporting of failure has but little of encouragement, and gladly turns to other States where more persistent and vigorous effort has met deserved success.

The State Board of Health of Illinois is now in its sixth year of successful operation, and as this Board is the pioneer, so far as is known to your committee, it is thought that a brief reference to its plan of organization and work accomplished may not be without interest and encouragement.

This Board exists, by and under the laws of the State, since June 1, 1877, and consists of seven members appointed by the governor, who hold office for seven years. As now constituted there are on the Board two lawyers, three physicians, one homœopathic physician and one eclectic physician. Besides the usual duties pertaining to Boards of Health, this Board has the duty of enforcing the medical practice act of the State, under which it grants certificates, the holders of which are entitled to practice medicine in the State.

Such certificates are granted to graduates of medical schools in good standing, and to persons practicing in the State for ten years prior to the passage of the act, and to non-graduates after passing a satisfactory examination made by the Board.

The Board has also the power to declare what is unprofessional conduct and to revoke certificates in cases of parties guilty of such conduct. One of the most important of the powers of this Board is that by which it declares what medical schools are in good standing—that is, what diplomas give their holders the right to ask for license to practice medicine in the State of Illinois without further examination from the Board. For the information of those interested, the Board have fixed and announced a minimum standard of requirements for colleges wishing to be considered in "good standing." This requirement provides for, in addition to the usual conditions, a preliminary examination in the branches of a good English education, efficient instruction in hygiene and medical jurisprudence, besides the eight ordinary Chairs, a twenty weeks' course, two courses not in the same year, attendance upon at least eighty per cent. of all the lectures in the various courses, regular examinations by each teacher twice weekly, final examinations, to be conducted by competent examiners other than the teacher in each branch, two courses of dissection, and two terms of clinical and hospital instruction, three full years of study with preceptor; and, more important than all the rest, the school must show that it has a corps of competent teachers and ample facilities to carry out the above in a thorough and efficient manner.

In making this announcement, the Board regrets that it cannot adopt as the minimum standard that required by the best schools of the country, but recognizes that reforms in medical schools must be gradual. This Board reports annually to the American Medical Association, the physicians upon the Board being members of that association in good standing. From the last report to the association in print I gather the following statistics of this portion of the work:

When the Board began its work, there were in the State 7,400 doctors of all sorts,; of them only about 3,600 were graduates or licentiates of medicine. In 1882, instead of 3,800 non-graduates, there were only 935, and in 1883 less than 300, and most of them were exempt from prosecution by the ten-year practice clause. Nearly 3,000 irregular practitioners have been driven from the State, and the proportion of inhabitants to physicians has risen from 500 to 600 to one, to 800 or 900 to one. In 1882 charges of unprofessional conduct against physicians have been investigated in thirty-nine cases and licenses revoked in eighteen cases. The Board has rejected the diplomas of eighteen medical colleges as not in good standing, and there are forty-five more not yet passed upon, but which the Board thinks will show about the average per cent. of poor ones.

The Board makes full report quarterly and annually, and altogether shows an amount of work done most interesting in character and remarkable in extent.

An enthusiastic speaker, at the recent meeting of the A. M. A., said that this Board had done more to elevate the standard of medical education and efficiency in its short term of six years than the association had accomplished during the last quarter of a century.

During the last year the States of Missouri, Minnesota and Delaware have established similar Boards, the laws of the first two States being almost identical with that of Illinois, while that of the latter is quite similar. West Virginia also has a Board of Examiners and license, but this consists of physicians only.

Your committee deem this statement not unworthy the attention of this society, not alone for the remarkable results accomplished by the Illinois Board, but more particularly from the new and important principle involved in the formation of this and similar Boards. It is plainly to be seen that a Board to do the work done by that of Illinois must be endowed by law with full powers, and that no sectarian body is at all likely to receive such endowment. Such a body must represent what the people of the State consider to be the medical profession.

Your committee believes that this work should be continued, to the end that there may be presented to the next session of the legislature a bill similar to the one recently recommended by the society, that advantage be taken of the time between now and the end of the year to confer with the different medical societies in the State, with prominent citizens and public persons with a view of securing their co-operation in assisting the passage of this proposed bill.

To this end your committee would offer and recommend the adoption of the following resolutions:

THE RESOLUTIONS.

*Resolved*, That it is the belief of this society that the present method of training and licensing physicians does not command the entire confidence of the profession or of the people.

*Second*—That the system of licensing physicians by a State Board of Examiners, representing the legally authorized medical profession, as seen in operation during the past six years in the State of Illinois, is a decided improvement upon that now pursued in this State.

*Third*—That this society hereby endorses the principle seen in the formation of mixed boards of examiners, that is, boards giving in one body *pro rata* representation to all the different schools of legally authorized physicians, and believes that it is through the agency of such boards that the standard of medical education may be most efficiently advanced, the profession most advantageously controlled and its ranks recruited by the admission only of persons worthy of its high responsibilities.

*Fourth*—That this society hereby pledges its best ability and endeavor to the cause of securing to the people of this State the services of such a board of examiners.

*Fifth*—That to this end a committee of seven members be appointed at this meeting to be known as the committee on legislation, and that this committee have full charge of this matter with power to call special meetings of this society, to confer, in the name of this society, with other societies and with individuals upon matters germain to this subject, to draw orders upon the treasury of this society in sums not to exceed $100 for all legitimate expenses incurred in the prosecution of this work, and with such other power as rightly used would, in its judgment, aid to bring about this greatly desired end.

The third resolution met with considerable opposition. The idea of a "Mixed Board" was not received favorably by many of the older members present. It was warmly discussed and finally carried by a very close vote, thirty-two to thirty.

In accordance with the resolutions, the Chair appointed, as a committee on legislation, Drs. John Hauenstein, W. D. Mann, F. S. Crego, Edward Storck, A. R. Davidson, H. R. Hopkins, A. H. Briggs.

Doctor Storck, chairman of the Board of Censors, presented the following report, which was received and the recommendations adopted:

In accordance with a resolution passed at a former meeting of the society, directing the Board to prepare a list of names of physicians practicing in the county, who have failed to register in the County Clerk's office, also the names of persons practicing without diploma or license, and prosecute such persons before a proper tribunal, the Board, on complaint of the president of the society, brought three cases before the police court, to be tried under the new penal code of the State, with the following result:

The first, Louis Beyerman, an ignorant quack, who had resided in Buffalo but a short time, through advertisements acquired a large practice and extorted large sums of money from his patients, and through malpractice, in several cases, had made himself guilty of manslaughter, plead guilty of the charge and was fined $50.00 by Judge King, who gave him the advice to leave town forthwith.

The second, Dr. Evans, a traveling doctor, who advertised himself as a *magnetic-healer;* he had registered a diploma of an Ohio medical college, and claimed to posess another one of Philadelphia. After he had received considerable money of his confiding patients, he was taken before Justice King, on a charge of practicing under cover of a diploma fraudently obtained. He was granted an adjournment of several days, but when the case was called again, the magnetic-healer had taken to his heels, leaving his security, $200.00 in cash, for the benefit of the city treasury.

In the third case, the defendant plead guilty of the charge of dealing out medicines, but as he claimed not to be a doctor and promised not to attempt to practice hereafter, Judge King suspended sentence, with the advice: to go and sin no more.

After your Board, together with the Censors of the Erie County Homœopathic Medical Society, had examined the registry and prepared a list of those who are thought to practice illegally in this county, which was partly published by the daily press, deferred further action and postponed the prosecution of such persons, until the question was decided whether an act passed by the last legislature by which a certain institution in this city that pretends to operate as a medical college under a charter twice invalidated by decisions of the courts was to be legalized and the incorporation made valid, would become a law by receiving the signature of the governor.

It would have been a useless task for your Board to proceed against these persons, practicing in violation of the law, many of whom are on the list of matriculants of said medical college, and would have been graduated and made legal practitioners in a very short time. Your Board would have asked the society to be discharged from further duties in this direction.

After having received official notice that the governor had not sanctioned the law as passed by the legislature, the Board resumed its labors, and together with the Censors of the other county medical society, decided to carry the law into effect against all illegal practitioners. A notice to this effect, together with a copy of the respective sections of the law and penal code of the State, will be addressed to all persons whom the Censors have reported at their last meeting, and prompt prosecution instituted against those who practice without having complied with the requirements of the law.

Your Board of Censors would ask of the society to be empowered to employ legal counsel and to take such action as may be necessary to restrain any illegal medical college in this county from issuing diplomas; also, ask for the necessary funds to carry the action through.

EDWARD STORCK, *Chairman.*
ANDREW D. MANN,
A. H. BRIGGS,
F. H. HOYER,
*Board of Censors.*

A vote of thanks was extended to the Board of Censors for the energetic and faithful manner in which they had thus far carried out their work. The Treasurer was directed to pay, on the order of the President of the society, amounts not to exceed one hundred dollars to defray the expenses of the Board.

Dr. P. W. Van Peyma being absent in Europe, Dr. H. R. Hopkins was appointed to act on the Board of Censors in his place.

At the afternoon session, Dr. Henry Nichell read an extremely interesting and practical paper regarding the code of ethics of the State Medical Society. The paper received the hearty endorsement of Professor Rochester, and all of the members present.

A vote of thanks was tendered the doctor, and a copy of the paper requested for publication in the BUFFALO MEDICAL JOURNAL.

On motion the society adjourned.

A. M. BARKER, *Secretary.*

